# Challenges associated with implementing anti-doping policy and programs in Africa

**DOI:** 10.3389/fspor.2022.966559

**Published:** 2022-12-08

**Authors:** Jonathan Ruwuya, Byron Omwando Juma, Jules Woolf

**Affiliations:** Department of Recreation, Sport and Tourism, University of Illinois Urbana-Champaign, Champaign, IL, United States

**Keywords:** African sport, clean sport, neo-colonialism, sport governance, sport policy

## Abstract

Concerns regarding the capability of the International Olympic Committee to address doping in sport catalyzed the formation of the World Anti-Doping Agency (WADA) in 1999. In its establishment phase, WADA sought geopolitical legitimacy and support from governments (including non-Western states) for financing and acceptance. Africa was not considered during WADA's creation, relegating African states to a passive role in the global anti-doping program, and yet is still subject to the strict compliance requirements for WADA's global policy. African countries face challenges establishing anti-doping support structures and implementing the universal policy, including competing macro-level policy demands that favor addressing legacies of colonialism and human capacity development. To develop robust anti-doping support structures, African nations must spearhead anti-doping initiatives by leveraging existing infrastructure and encouraging collaborations between NADOs such that capacity can be built for policy implementation.

## Introduction

On November 5, 1998, the members of the Monitoring Group of the Council of Europe's Anti-Doping Convention held an extraordinary meeting in Strasbourg, France, to prepare proposals for the 1999 World Conference on Doping in Sport and recount recent events in the doping sphere. This included discussions on the Festina affair in France where large quantities of the endurance-enhancing drug erythropoietin were uncovered by custom agents on route to a professional cycling team, the case of Chinese swimmers' attempted effort to bring human growth hormone into Australia during the World Swimming Championships, and deficient doping control procedures in Italian football (1). In sum, there were concerns that doping was out of control and would erode public confidence in sport. Though the Convention was open to signatories outside Europe, only two non-European States (Canada and Australia) had signed, out of a total of 41 countries, by the end of 1998 (2). While South Africa honored the invitation, no other African country was invited. Though the Supreme Council of Sport in Africa was enjoined in the development of WADA after the conference, like most non-European countries, Africa's input was limited in implementing the conference's recommendations. Such developments suggest that the African perspective was not considered during the anti-doping policy formation processes. Yet, there are strict compliance requirements to mandatory aspects of a seemingly Western-European formulated anti-doping policy, to which African signatories to the WADA Code (the rules that harmonize anti-doping policy globally) are bound. It is salient to mention that academic studies of anti-doping policy have similarly been dominated by a Western-European perspective. Therefore, we contend that it is critical to understand anti-doping policy practices from other regions, in our case, Africa. In this policy brief article, we discuss Africa's peripheral role in forming WADA and the potential impact on anti-doping policy acceptance in the continent. We further examine issues that position sport initiatives in Africa as peripheral and illuminate the challenges associated with establishing anti-doping support structures and implementing anti-doping policy and programs. In doing so, we argue that the slow progress in developing vigorous anti-doping programs in Africa is due to neo-colonial factors and African governments' priorities that render sport and anti-doping initiatives inconsequential, perhaps a perspective underappreciated by Western-European counterparts. Actionable recommendations to build anti-doping capacity through leveraging existing structures and establishing collaborations with various actors are provided.

## Africa's peripheral role in the creation of WADA

The failure to adequately incorporate Africa in the activities preceding the formation of WADA was myopic and arguably led to decisions that undermined WADA's legitimacy in Africa. Executive decisions to create WADA were made predominantly from a western-centric standpoint (3), yet compliance was sought beyond Europe. This fueled the notion of neo-colonialism, especially in Africa. This apparent imposition of the Eurocentric version of sport (including anti-doping) may be perceived as cultural imperialism and colonization (4). African states had their unique experiences with sport which they participated in long before colonization. For example, wrestling was popular in Africa with organized intervillage or interkingdom competitions accompanied by various rituals officiated by elders (5). These physical contests were important social events and drew audiences from miles around with male competitors training with fathers and other close relatives far in advance in hopes of “throwing” their opponents and, thus, being deemed an asset to family and community (6). Wrestling was central to the acquisition of manhood (6) and developed to the extent that makes it comparable with the Olympic Games (7).

Though the organization and governance of modern sport might seem foreign, the nature of activities engaged in are not any different from those of the pre-colonial generation. Performance and winning competitions were of utmost importance in both instances. Yet, ill-will had no place and credit was given for the exhibition of fairness (5). To gain credit for their kin-group, wrestlers sometimes took to “doping” by swallowing stimulants both before and during the match (5). Such practices were not against the “Spirit of sport” but were in fact part of the sport's milieu. Indeed, when anti-doping was introduced in Senegal, West Africa, in 2015, a local wrestling champion, Sa Thies equated the imposition of doping control as an attempt to modernize wrestling, when he stated, “We don’t need doping tests in our sport. Wrestling isn’t a white man's business” (8). Beyond wrestling, other sports popular in Africa had practices that challenged Western and Eurocentric conceptualization of doping. For instance, the use of “jujus” and “jars” (e.g., talismans) was a common and revered form of spiritual doping that was prevalent in football in West Africa (9). While these practices would not violate anti-doping rules, by not contemplating or addressing such deeply embedded cultural beliefs the legitimacy of WADA, from an African perspective, may have been called into question. In effect, disregarding the African context led to anti-doping policies that, without explanation, simultaneously outlawed practices not considered doping while ignoring others that were thought performance-enhancing. Such sentiments demonstrate the folly of not incorporating the African perspective in anti-doping.

However, WADA has sought other strategies to gain acceptance in Africa. WADA's need for geopolitical legitimacy during its formation phases, instigated the establishment of a regional office in Cape Town, South Africa (10). The office was to educate and convince governments about the importance of a harmonized global approach to preventing doping in sport. The establishment of the regional office was crucial to eliminating neo-colonial perceptions of a Western-led global anti-doping program from African leaders. Additionally, the funding model adopted by WADA sought to reduce the financial burden on the region and consequently gain acceptance. The Olympic Movement and Governments of the world equally fund WADA's core budget. As per the Copenhagen Declaration, the African region is expected to contribute 0.5% of the governments' share (11). Despite the lower financial expectation, the contribution from the region has been inconsistent. Africa contributed 64% (12) and 44% (13) of their share in 2021 and 2022, respectively. To further gain the trust of African leaders, WADA facilitated the creation of five Regional Anti-Doping Organizations (RADOs) in 2004 to specifically support anti-doping policy implementation in less-resourced National Anti-Doping Organizations (NADOs) and National Olympic Committees (NOCs) through funding, training, and ongoing anti-doping assistance. These RADOs are led by individuals within the region, therefore allowing these regions to administer their anti-doping programs. At the national level, NADOs are expected to fund their operations. Article 23.3 of the Code requires signatories to the Code (e.g., NADOs) to devote sufficient resources to implement anti-doping programs (14). However, as we shall demonstrate in this policy brief, there are competing needs that governments in developing countries deem essential. Unfortunately, sport is not one of them.

These efforts to support anti-doping programs in Africa by WADA are seemingly instrumental in gaining geopolitical legitimacy. Furthermore, African governments support the world anti-doping program, as shown by 52 African countries that have ratified the International Convention against Doping in Sport (15). To date, 25 of 54 African countries have operational NADOs, and all these organizations were established within the last ten years. Also, WADA has incorporated African states in its current governance structure (16). The WADA President, Witold Bańka, has recognized the need to build capacity across the African continent such that each nation has its own robust anti-doping program (17). However, such efforts need to be contextualized in the history of colonialism experienced in Africa.

## The brunt of colonialism: limited resources and competing needs

The developmental processes experienced by African states, which are associated with colonialism and the subjugation of African states by European imperialists, have created circumstances where sport policy and programs, in general, and anti-doping policy can be fringe. Colonialism is regarded as a practice by one people or political power of obtaining full or partial control over another people or political system (country), occupying the controlled state with settlers, and exploiting the controlled people and space economically (18). In Africa, colonialism, which took place between 1880 and 1935, was signified by white hegemony that elicited social stratifications and benefited the white minority colonists (19). The governance structures founded on white hegemony ensured that the subordinate autochthons were disadvantaged politically, socially, and economically. In the wake of Pan-Africanism and revolutionary rhetoric, many African states attained independence from their colonizers, inheriting colonial legacies (e.g., poor education for black people, poverty, poor living conditions, unemployment for the youth, and vast wealth and social inequality) requiring urgent attention.

From a public policy perspective, social issues that are regarded as critical receive attention from policymakers and the public. Social issues become part of government policy agenda through a series of discussions by policymakers and social indicators that draw attention to the need to address the matter through policy intervention (20). Likewise, the importance of colonial reparations in an African context (e.g., providing clean water, housing, electricity, education to black Africans, and public health) influences government budgetary allocations, largely limiting financial resources to sport-related programs. The larger socio-economic issues associated with the colonial legacy and a nation's economy are, therefore, competing needs that inform overall government priorities which limits attention to sport (21). For example, sport for development initiatives in African countries such as Tanzania struggled to gain attention or resources as these efforts have had to contend with the nation's key policy priorities, such as addressing extreme poverty and attempting to develop *via* tourism and trade (22).

The limited funding to sport presents challenges to implementing, monitoring, and evaluating sport policies and programs (23). Furthermore, Ruwuya (21) has reported that critical issues in African sport (e.g., corruption, competition manipulation, human rights abuses including sexual abuse, and age cheating) other than doping, concern policymakers. Consequently, the scant resources in sport are also used to address these issues. With limited funding, critical issues in sport are prioritized, and as discussed, those issues that resonate with the public and policymakers (e.g., sexual abuse) are more likely to be placed higher up on the policy agenda. Moreover, the shortfall is left to a sport industry that is nascent and incapacitated. The result is the impeded development of robust sport programs, including doping programs, and inadequate implementation, monitoring, and evaluation of sport-related policies and programs (22, 23).

Limited financial resources, additionally, hinder the establishment of anti-doping support structures and systems. By signing the UNESCO Convention against Doping in Sport, State Parties committed to supporting the world anti-doping program in their countries. The commitment to support the anti-doping program has a financial burden, especially concerning establishing an independent NADO responsible for administering anti-doping programs (doping control and testing programs, anti-doping education programs, and anti-doping research) in a country. For example, doping control and testing is a central tenet of the universal anti-doping program (although WADA has recognized the importance of anti-doping education showcased by the development of the International Standard for Education), however, it is expensive for nascent NADOs to implement adequately. Consequently, International Federations (e.g., World Athletics through the Athletics Integrity Unit) continuously fund anti-doping testing initiatives for under-resourced NADOs in Africa to ensure the adequate testing of athletes.

While 52 African countries ratified the UNESCO Convention against Doping in Sport, only 25 African nations have established independent NADOs within the last decade. Although this signifies a wider acceptance of the universalist anti-doping program and growth of anti-doping work in Africa, the delayed progress can be linked to the overall government objectives to address inequalities and broader social issues as legacies of colonialism, as aforementioned. Additionally, the absence of the experienced personnel needed to develop the necessary support structures to run such programs further hinders the efforts to establish strong anti-doping programs.

## Capacity and anti-doping programs in Africa

There are limited opportunities to study sport and related areas in Africa. Consequently, most African countries have developed little human resource capacity in sport management. This lack of expertise presents challenges for anti-doping policy implementation, education programs, and management of sport in general. This means that sport has largely remained intellectually starved, with the running of sport left to people who lack relevant training, possibly government-appointed, and serve limited terms leading to high turnover and a loss of institutional knowledge (21). For example, Mwisukha and Mabagala (24) note that most personnel serving in various national federations and organizations in East Africa lack adequate sport management skills. Such limited human capacity negatively impacts service delivery in sport and sport development (25). Even when trained staff are available, they may only work part-time, which has implications for the reach and depth of anti-doping programming. For instance, Juma and colleagues (26) observed that many anti-doping educators in Kenya worked part-time and their time with athletes was fleeting. While elite athletes received support, most Kenyan athletes had limited to no access to education. However, the Anti-Doping Agency of Kenya (ADAK) took a novel approach. It partnered with the Kenya Institute of Curriculum Development (KICD) to roll out a values-based education program to promote positive sporting values among school-going children. Such efforts demonstrate that implementing anti-doping policy (such as education requirements) can be achieved using existing structures to overcome the constraints that African anti-doping policymakers and administrators encounter.

## Actionable recommendations

For African nations to develop robust anti-doping programs, they may need to spearhead anti-doping initiatives within the continent and encourage partnerships and collaborations between NADOs in Africa to build capacity. African nations may also need to pursue partnerships between NADOs/RADOs and corporations, as well as partnerships with other government departments and agencies to counter resource inadequacy. We, therefore, propose the following recommendations.

First, in addition to WADA's efforts to assist and support African nations to establish vigorous anti-doping programs by establishing the RADOs and the WADA office in South Africa, there is a need for African countries to spearhead anti-doping efforts in the continent. A case in point is health research in Africa, where there has been a shift from international-led research and capacity building towards more Africa-led models (27). This move has been deemed important by scholars, such as Izugbara et al. (28), since Africans are better placed to understand, articulate and prioritize their social issues. Though support from other partners outside of Africa is important, we agree with Kasprowicz and colleagues (27) that capacity within the continent will not improve unless African countries take a leading role. When African countries initiate research activities, the outcomes can be improved. This is evident in African-led initiatives in health research which have led to improved research outputs (29), relevant topics and communication of findings in cultural and policy contexts (30, 31).

To achieve this, greater demand for human capital in the region is needed. Some efforts in this regard are taking place, as shown by the NBA's movement into Africa (32) and partnerships between European and African Football Clubs (33). However, more opportunities exist. For example, International Federations and major sporting organizations could award more events to African states. The regular hosting of events could spur interest in sport, leading to greater and sustainable demand for skilled workers. Such efforts would need to be enacted with caution so that this does not lead to an undue financial burden on the host country (34). However, smaller events, such as the 2017 IAAF World U18 Championship and the 2021 World Athletics U20 Championships, have been successfully hosted in Nairobi, Kenya, and the 2026 Summer Youth Olympic Games will take place in Dakar, Senegal. This demonstrates African countries' capability to host international sporting events. Developing demand for trained workers to manage and organize sport would also benefit anti-doping organizations in the region. Such strategies (i.e., the development of African-led sport and anti-doping administration) would spur acceptance of programs among athletes and stakeholders alike as these might not be deemed foreign. Thus, the development of human capital would enable African countries to lead anti-doping efforts within the continent, while fostering ownership and encouraging Code compliance.

Second, partnerships and collaborations between NADOs in Africa offer great opportunities for capacity building through information sharing and technical training based on mutual understanding and appreciation of socio-cultural factors. WADA's President, Witold Bańka has previously underscored the importance of collaboration in building anti-doping capacity across the continent of Africa (17). With the paucity of research in sport in Africa, policy implementation and partnerships could borrow from existing frameworks in other sectors such as food and health. In their research on policy partnerships on mental health and poverty projects in Ghana, South Africa, Uganda, and Zambia, Mirzoev et al. (35), identified the principles of trust, openness, equality, and mutual respect as the core of partnerships with individual, organizational and contextual factors influencing its success. In forging these partnerships, organizations should make efforts to ensure that the personal expectations and perceived benefits of the individuals involved are realistic and achievable. In addition, roles and responsibilities including the internal structures of these partnerships should be non-bureaucratic, formalized, and clearly stipulated in a Memorandum of Understanding. Also, of importance to the success of these partnerships are contextual factors such as political goodwill. African states should appreciate the rich history of sport in Africa and the impact it can have when leveraged (5). Consequently, African governments should foster environments where sport thrives, ensuring the growth of these partnerships.

After the creation of WADA, African states looked up to established NADOs in Europe and Asia for technical support as they sought to operationalize their NADOs. Apart from South Africa, there was no country in Africa which had the technical knowhow of anti-doping. When the ADAK launched its program, it relied heavily on NADOs from England, China, and Norway to develop the technical competencies and educate its staff (36). As countries in Africa develop their anti-doping technical competencies, partnerships between NADOs in Africa are desirable. The partnerships between Kenyan NADO and Ghana (37), and South African and Ethiopian NADOs (38) are cases in point. Both partnerships seek to give developing NADOs access to the resources and experience of more developed and experienced NADOs. However, capacity building involves more than formal training and individual development but rather a long-term process requiring lasting mentorship, coaching, and leadership development, including an individual commitment to continued self-development (39). The NADO-NADO partnerships should have a long-term engagement strategy based on mutual and beneficial objectives for all parties involved to maintain enthusiasm and continued engagement. Conferences, workshops, and symposia offer information-sharing opportunities which African NADOs can leverage to share information on best practices. An annual or bi-annual conference where African countries meet to share their experiences on anti-doping would spur the development of good practices, knowledge generation, sharing, and acquisition.

Third, NADOs could leverage the existing infrastructure and collaborate with other government agencies to run anti-doping programs. Government agencies such as departments of Health, Education or Social Services are ideal partners in anti-doping education. The ADAK-KICD partnership mentioned earlier provides an exemplar case. Resources too can be acquired through collaboration. Partnerships between NADOs and corporations could plug the budgetary deficit experienced by most NADOs. These additional resources could be used for anti-doping programming such as capacity development of staff and athletes' education. In the former case, this could eventually lead to the increased attraction of sport management as a career choice. Regional and continental partnerships for anti-doping purposes should be pursued by the RADOs, and the WADA Regional Office with the help of WADA. Recently, WADA signed a sponsorship agreement with SuperSport (African-based broadcaster) that will provide direct funds and in-kind value to promote and coordinate the protection of clean sport in Africa (40). Though this is a positive development, WADA's regional office in Cape Town should play the leading role in the operationalization of the partnership. This will serve to limit the perception of western influence on African states. Additionally, the IOC and WADA should set aside resources which African nations could tap into to support the development and operationalization of NADOs.

## Conclusion

In this policy brief article, we have historically situated the challenges of implementing anti-doping policy and programs in Africa. These challenges have been associated with the peripheral role Africa played in the creation of WADA, and the colonial legacies that governments prioritize over sport, limiting financial resources and human capacity in sport. Though Africa continues to address inequalities and other social issues linked to colonialism, we have provided recommendations that may assist building robust anti-doping programs, and aid anti-doping policy implementation in the continent. The resource and capacity inefficiencies notwithstanding, African states are taking steps to implement anti-doping policy. As such, these countries should be supported in developing their NADOs.
